# Feasibility of Using Real-World Data to Replicate Clinical Trial Evidence

**DOI:** 10.1001/jamanetworkopen.2019.12869

**Published:** 2019-10-09

**Authors:** Victoria L. Bartlett, Sanket S. Dhruva, Nilay D. Shah, Patrick Ryan, Joseph S. Ross

**Affiliations:** 1 Yale School of Medicine, New Haven, Connecticut; 2 Department of Medicine, University of California, San Francisco School of Medicine, San Francisco; 3 Section of Cardiology, San Francisco Veterans Affairs Health Care System, San Francisco, California; 4 Division of Health Care Policy & Research, Mayo Clinic, Rochester, Minnesota; 5 Epidemiology Analytics, Janssen Research and Development, Titusville, New Jersey; 6 Observational Health Data Sciences and Informatics (OHDSI), Department of Biomedical Informatics, Columbia University Medical Center, New York, New York; 7 Section of General Internal Medicine and the National Clinician Scholars Program, Yale School of Medicine, New Haven, Connecticut; 8 Department of Health Policy and Management, Yale School of Public Health, New Haven, Connecticut; 9 Center for Outcomes Research and Evaluation, Yale-New Haven Hospital, New Haven, Connecticut

## Abstract

**Importance:**

Although randomized clinical trials are considered to be the criterion standard for generating clinical evidence, the use of real-world evidence to evaluate the efficacy and safety of medical interventions is gaining interest. Whether observational data can be used to address the same clinical questions being answered by traditional clinical trials is still unclear.

**Objective:**

To identify the number of clinical trials published in high-impact journals in 2017 that could be feasibly replicated using observational data from insurance claims and/or electronic health records (EHRs).

**Design, Setting, and Participants:**

In this cross-sectional analysis, PubMed was searched to identify all US-based clinical trials, regardless of randomization, published between January 1, 2017, and December 31, 2017, in the top 7 highest-impact general medical journals of 2017. Trials were excluded if they did not involve human participants, did not use end points that represented clinical outcomes among patients, were not characterized as clinical trials, and had no recruitment sites in the United States.

**Main Outcomes and Measures:**

The primary outcomes were the number and percentage of trials for which the intervention, indication, trial inclusion and exclusion criteria, and primary end points could be ascertained from insurance claims and/or EHR data.

**Results:**

Of the 220 US-based trials analyzed, 33 (15.0%) could be replicated using observational data because their intervention, indication, inclusion and exclusion criteria, and primary end points could be routinely ascertained from insurance claims and/or EHR data. Of the 220 trials, 86 (39.1%) had an intervention that could be ascertained from insurance claims and/or EHR data. Among the 86 trials, 62 (72.1%) had an indication that could be ascertained. Forty-five (72.6%) of 62 trials had at least 80% of inclusion and exclusion criteria data that could be ascertained. Of these 45 studies, 33 (73.3%) had at least 1 primary end point that could be ascertained.

**Conclusions and Relevance:**

This study found that only 15% of the US-based clinical trials published in high-impact journals in 2017 could be feasibly replicated through analysis of administrative claims or EHR data. This finding suggests the potential for real-world evidence to complement clinical trials, both by examining the concordance between randomized experiments and observational studies and by comparing the generalizability of the trial population with the real-world population of interest.

## Key Points

**Question:**

What percentage of clinical trials published in high-impact journals in 2017 generated evidence that could feasibly be replicated using observational methods and data sources?

**Findings:**

In this cross-sectional study of 220 clinical trials published in high-impact journals in 2017, only 15% could feasibly be replicated using currently available real-world data sources.

**Meaning:**

This study suggests that, although the increasing use of real-world evidence in medical research presents opportunities to supplement or even replace some clinical trials, observational methods are not likely to obviate the need for traditional clinical trials.

## Teaser

This cross-sectional study analyzes the feasibility of replicating clinical trials by examining whether the interventions, indications, inclusion and exclusion criteria, and primary end points could be ascertained from observational data from insurance claims and/or electronic health records (EHRs).

## Introduction

Randomized clinical trials (RCTs) are generally considered to be the criterion standard for generating clinical evidence. Randomizing patients to different treatment arms, specifying inclusion and exclusion criteria, and ascertaining patient outcomes through follow-up visits enhance the internal validity of a trial. However, these features may contribute to the RCT’s lack of generalizability to real-world clinical practice.^1^ These trials are also expensive^2^ and take a long time to complete. These shortcomings are increasing the interest in alternative approaches to evaluating the efficacy and safety of medical interventions.^3^

Real-world data are defined by the US Food and Drug Administration (FDA) as “data related to patient health status and/or the delivery of health care routinely collected from… electronic health records (EHRs), claims and billing data, data from product and disease registries, patient-generated data including home-use settings, and data gathered from other sources that can inform on health status, such as mobile devices.”^4^^(p4)^ Real-world data are analyzed to create real-world evidence (RWE), which is clinical evidence about “the usage, and potential benefits or risks, of a medical product derived from analysis of RWD.”^4^^(p4)^ Compared with RCTs, RWE better reflects the actual clinical environments in which medical interventions are used, including patient demographics, comorbidities, adherence, and concurrent treatments.^5,6,7,8^ The 21st Century Cures Act of 2016, a harbinger of the increasing use of EHR and insurance claims data for medical research in the United States, required the FDA to develop guidance on the use of RWE in studies of medical product safety and outcomes for both postapproval studies and studies of new indications of approved drugs.^4,9^

Broader availability of EHR and other clinical data, in combination with advancements in statistical methods and computing capacity, may allow researchers to increasingly use observational methods to retrospectively generate RWE. Although the Federal Food, Drug, and Cosmetic Act does not require that substantial evidence be based on RCTs to support drug approval,^10^ enthusiasm for using retrospective RWE to complement the evidence generated by RCTs may still need to be tempered by feasibility concerns because it is unclear whether it is reasonable to expect that observational data can be used to address the same clinical questions being answered by traditional clinical trials. Accordingly and to inform contemporary expectations for RWE, we conducted a cross-sectional study of clinical trials published in high-impact journals in 2017. Our aim was to examine whether the evidence generated could be feasibly replicated using observational data available in the United States from EHRs or insurance claims.

## Methods

### Data Source and Study Sample

Because this cross-sectional study did not involve human participants and used publicly available data, it did not require review and approval by an institutional review board or ethics committee, according to the Yale Human Investigation Committee. No informed consent was obtained given that the data used did not contain any identifiable information and participants could not be contacted. This study followed the Strengthening the Reporting of Observational Studies in Epidemiology (STROBE) reporting guideline.

We identified the top 7 high-impact journals in the category of medicine, general and internal, defined as journals with an impact factor greater than 10 in 2016 as rated by Web of Science’s Journal Citation Reports.^11^ The 7 journals were *New England Journal of Medicine, Lancet, JAMA, The BMJ, Annals of Internal Medicine, JAMA Internal Medicine,* and *PLoS Medicine.* One of us (V.L.B.) searched PubMed to identify all prospective clinical trials regardless of randomization that were published in these 7 journals by using clinical trial as the article type and the period from January 1, 2017, to December 31, 2017, as the publication date range. Any uncertainties were clarified with another one of us (J.S.R.).

We excluded trials that did not involve human participants; did not use end points that represented clinical outcomes among patients (eg, trials with physician prescribing as an end point); were not characterized as a clinical trial by the authors, including those characterized as a cohort or registry-based study; and had no recruitment sites in the United States given that EHR systems and insurance claims structures may differ outside the United States and we have limited experience using observational data from non-US sources.

### Main Outcome Measure

The primary outcome was the number and percentage of trials in which an intervention, indication, inclusion and exclusion criteria, and primary end point could be ascertained from insurance claims and/or EHR data. One of us (V.L.B.) systematically reviewed each trial to establish whether each of its characteristics was likely to be routinely ascertained from structured EHR data and/or insurance claims data. All determinations that the information could not be routinely ascertained were reviewed and confirmed by another one of us (J.S.R.).

First, we examined the studied intervention, as specified in the published article’s abstract. Interventions that were considered not likely to be routinely ascertained from observational data included drugs or biological agents that had not yet been FDA approved because they lacked a National Drug Code; medical devices, given the lack of adoption of the Unique Device Identifier^12^; and educational, behavioral, and procedural interventions that lacked specific *International Classification of Diseases, Ninth Revision* (*ICD-9*) procedure codes, *International Statistical Classification of Diseases and Related Health Problems, Tenth Revision* (*ICD-10*) procedure codes, or *Current Procedural Terminology* codes or that required additional clinical information not routinely available, such as use of a decision aid or treatment algorithm. If the trial included a placebo group as a comparator to the intervention group, this information was not taken into account when establishing the feasibility of ascertaining the intervention from observational data.

Second, we examined the indication or condition being treated, as specified in the published article’s abstract. Indications that were considered not likely to be routinely ascertained from observational data included those for which researchers would find it difficult to develop a computable phenotype using available methods and sources (ie, structured EHR data or claims data). For example, if eligible patients were identified on the basis of an indication qualifier, such as active psoriatic arthritis, or a severity determination, such as moderate-to-severe plaque psoriasis, this information was considered as not likely to be routinely ascertained from structured EHR or claims data.

Third, we examined the trial inclusion and exclusion criteria, identified from the trial registration information on ClinicalTrials.gov.^13^ Criteria that were considered not likely to be routinely ascertained from observational data were those that required data not found in structured EHR data fields, including pathologic results, most imaging results, physical examination findings, or other diagnostic tests that are not a routine part of the diagnosis and treatment protocols for a disease (eg, genetic testing). For a trial to qualify, we required at least 80% of the individual inclusion and exclusion criteria to be routinely ascertainable.

Fourth, we examined the trial’s primary end points, as specified in the published article’s abstract. End points were considered as not likely to be routinely ascertained from observational data if they required clinical information not found in structured EHR data or claims. For example, end points based on so-called hard clinical outcomes (such as diagnosis of incident condition), laboratory measurement, procedure use, other health care utilization (such as hospitalization), or overall survival were considered as routinely ascertainable from observational data. In contrast, end points based on functional disease scores, quality-of-life measures, or other patient-reported outcomes were not considered to be routinely ascertainable.

### Other Variables of Interest

For all trials, we characterized the type of intervention studied (eg, pharmaceutical; medical device; clinical or surgical procedure; educational, procedural, or behavioral; over-the-counter medication; and diagnostic or laboratory test), number of patients enrolled, and number of recruitment sites, including recruitment site location (eg, United States only vs United States and international, as well as clinical setting: hospital-based, primary care, specialty outpatient, skilled nursing, or multiple settings). Among studies characterized by the authors as clinical trials, we noted the use of randomization, allocation concealment, and a comparator arm (active, placebo, or none).

### Statistical Analysis

We used descriptive statistics to characterize the percentage of trials in which an intervention, indication, inclusion and exclusion criteria, and primary end point could be ascertained from insurance claims and/or EHR data. We used the Fisher exact test to examine statistical associations between trial characteristics and replicability, defined as having an intervention, indication, inclusion and exclusion criteria, and primary end point that could be ascertained from insurance claims and/or EHR data. Two-sided statistical tests, using a threshold of *P* = .05 for statistical significance, were used. Data were analyzed in Excel, version 14.1.3 (Microsoft Corp), and Stata, version 15.1 (StataCorp LLC).

## Results

In 2017, a total of 429 prospective clinical trials were published in high-impact general medical journals. We excluded 1 trial not involving human participants, 4 trials not involving clinical outcomes among patients, 7 trials characterized by the authors as cohort or registry-based studies, and 195 trials with no recruitment sites in the United States. The final sample comprised 220 trials (51.3%) (Figure).

**Figure.**
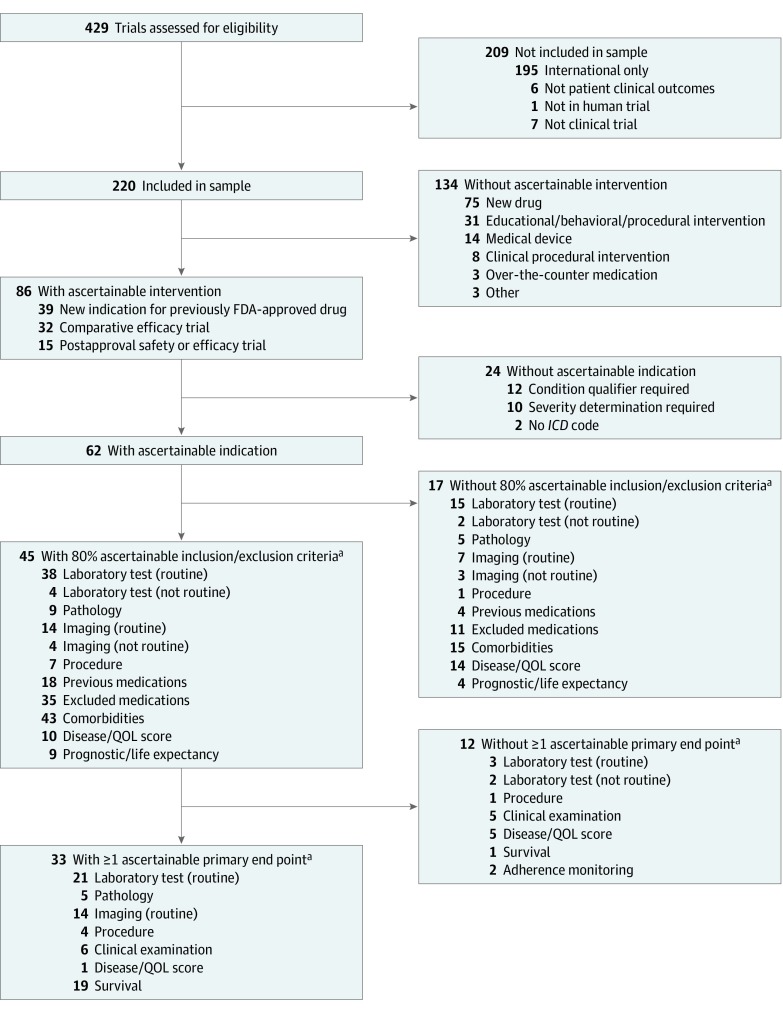
Study Flowchart Included are reasons that specific clinical trial characteristics could not be reliably ascertained from electronic health record or claims data. FDA indicates US Food and Drug Administration; *ICD*, *International Classification of Diseases*; and QOL, quality of life. ^a^Breakdown does not sum to the total because some trials had multiples of these criteria. Breakdowns count the number of trials with a specific characteristic and not the number of individual criteria or end points.

Of the 220 trials, 147 (66.8%) tested pharmaceutical interventions; 34 (15.5%) tested educational, procedural, or behavioral interventions; 20 (9.1%) tested clinical or surgical procedures; 14 (6.4%) tested medical devices; and 5 (2.3%) tested other interventions (Table).

**Table.** Characteristics of US-Based Prospective Clinical Trials Published in High-Impact General Medical Journals in 2017

Variable	No. (%)	Replicable, No. (%)	*P* Value^a^
Type of intervention^b^			<.001
Pharmaceutical product	147 (66.8)	24 (16.3)	
Seeking FDA approval	75 (34.1)	0	
Already FDA approved, seeking new indication	41 (18.6)	11 (26.8)	
Postapproval safety or efficacy	13 (5.9)	6 (46.2)	
Comparative efficacy	17 (7.7)	7 (41.2)	
Bioequivalence	1 (0.5)	0	
Medical device	14 (6.4)	0	
Educational, behavioral, or procedural intervention	34 (15.5)	0	
Clinical or surgical procedural intervention	20 (9.1)	7 (35.0)	
Other	5 (2.3)	2 (40.0)	
No. of study patients			.002
0-100	28 (12.7)	0	
101-500	76 (34.5)	7 (9.2)	
501-1000	50 (22.7)	9 (18.0)	
≥1001	66 (30.0)	17 (25.8)	
No. of recruitment sites			<.001
0-10	63 (28.6)	3 (4.8)	
11-50	51 (23.2)	4 (7.8)	
51-100	31 (14.1)	4 (12.9)	
101-200	42 (19.1)	11 (26.2)	
≥201	33 (15.0)	11 (33.3)	
Recruitment site location			.12
US only	90 (40.9)	9 (10.0)	
US and international	130 (59.1)	24 (18.5)	
Recruitment site clinical setting			.26
Hospital-based	82 (37.3)	12 (14.6)	
Primary care	15 (6.8)	1 (6.7)	
Specialty outpatient	13 (5.9)	0	
Other	8 (3.6)	0	
Multiple setting	41 (18.6)	6 (14.6)	
Not described/could not be determined	61 (27.7)	14 (22.9)	
Randomized			.48
Yes	204 (92.7)	32 (15.7)	
No	16 (7.3)	1 (6.3)	
Allocation concealment			.009
Double-blind	113 (55.4)	13 (11.5)	
Single-blind	30 (14.7)	2 (6.7)	
None	61 (29.9)	17 (27.9)	
Comparator			.19
Active	115 (52.3)	22 (19.1)	
Placebo	87 (39.5)	10 (11.5)	
No comparator	18 (8.2)	1 (5.6)	

Abbreviation: FDA, US Food and Drug Administration.

^a^ *P* values were calculated with Fisher exact test, which was used to examine the associations between trial characteristics and replicability.

^b^ Fisher exact test considered all pharmaceutical product types as a single category.

Among the 220 clinical trials, 28 (12.7%) enrolled 100 or fewer patients, 76 (34.5%) enrolled between 101 and 500 patients, 50 (22.7%) enrolled between 501 and 1000 patients, and 66 (30.0%) enrolled more than 1000 patients. In addition, 63 (28.6%) had 10 or fewer recruitment sites, 51 (23.2%) had between 11 and 50 recruitment sites, 31 (14.1%) had between 51 and 100 recruitment sites, 42 (19.1%) had between 101 and 200 recruitment sites, and 33 (15.0%) had more than 200 recruitment sites. Eighty-two trials (37.3%) recruited from hospital-based sites, 15 (6.8%) from primary care sites, 13 (5.9%) from specialty outpatient sites, 41 (18.6%) from multiple settings, and 8 (3.6%) from other settings, but the recruitment setting could not be identified for 61 trials (27.7%). Ninety trials (40.9%) had recruitment sites within the United States only, whereas 130 (59.1%) had trial sites located within and outside the United States.

Among the 220 clinical trials, 204 (92.7%) were randomized and 16 (7.3%) were not. Of the RCTs, 113 (55.4%) were double-blind, 30 (14.7%) were single-blind, and 61 (29.9%) were open-label. In addition, 115 (52.3%) of the 220 trials had an active comparator, 87 (39.5%) had a placebo comparator, and 18 (8.2%) had no comparator.

### Ascertaining the Trial Intervention and Indication

Of the 220 trials, 86 (39.1%) were determined to have an intervention that could be routinely ascertained from observational data. Commonly studied interventions that could not be ascertained from structured EHR or claims data were new drugs that had not yet secured FDA approval (75 trials [34.1%]); educational, behavioral, or procedural interventions (31 trials [14.1%]); medical device trials (14 trials [6.4%]); interventions that required additional clinical information, such as a Crohn disease management algorithm using clinical symptoms, and biomarkers that could not be ascertained from structured EHR or claims data (8 trials [3.6%]); over-the-counter medications (3 trials [1.4%]); and other trials (3 [1.4%]) for which interventions could not be ascertained. Of the trials for which interventions could be ascertained, 39 (17.7%) were for drugs already approved by the FDA drugs but for a new population or new indication, 32 (14.5%) were comparative effectiveness trials, and 15 (6.8%) were postapproval safety or efficacy trials (Figure).

Of the 86 trials for which the intervention could be ascertained, 62 (72.1%) had indications that could also be routinely ascertained from structured EHR or claims data. A common indication that could not be ascertained from EHR or claims data required indication qualifiers that did not have an *ICD-9* or *ICD-10* code and could not be found in structured EHR data (12 of 24 trials [50.0%]), required severity determination (10 trials [41.7%]), or involved an indication that did not have an *ICD-9* or *ICD-10* code, such as blunt torso trauma or acute extremity pain (2 trials [8.3%]) (Figure).

### Ascertaining the Trial Inclusion and Exclusion Criteria and Primary End Point

Among the 62 trials for which the intervention and indication could be ascertained, at least 80% of the inclusion and exclusion criteria data could be routinely ascertained from structured EHR or claims data for 45 trials (72.6%). Of the 17 trials that were below the 80% threshold, 14 (82.4%) required a subjective disease or quality-of-life score, 7 (41.2%) required routine imaging, 3 (17.6%) required a nonroutine laboratory test, and 5 (29.4%) required a pathological result that could not be ascertained from the structured EHR or claims data (Figure). Of the 45 trials for which the intervention, indication, and inclusion and exclusion criteria could be ascertained, 33 (73.3%) included at least 1 primary end point that could be routinely ascertained from structured EHR or claims data. Thus, of the 220 clinical trials published in high-impact general medical journals in 2017, 33 (15.0%) generated evidence that could be routinely ascertained from observational data (Figure). Rates of replicability stratified by journal are reported in the eTable in the Supplement.

### Association Between Study Characteristics and Replicability

Several trial characteristics were found to be associated with whether the trial could be feasibly replicated using currently available observational data from EHRs and insurance claims, including that trials with a larger number of enrolled patients (0% of trials with ≤100 patients vs 25.8% of trials with ≥1000 patients; *P* = .002) and recruitment sites (4.8% of trials with ≤10 sites vs 33.3% of trials with ≥200 sites; *P* < .001) were more likely to be replicable (Table). Among the trials of FDA-regulated pharmaceutical interventions, 24 of the 72 trials (33.3%) that already had received FDA approval were replicable compared with 0 of 75 that had not yet received FDA approval. However, other trial characteristics, including recruitment site type, randomized or blinded design, and comparator type, were not associated with the likelihood of being replicated.

## Discussion

In this study of US-based clinical trials with human participants that were published in high-impact journals in 2017, only 15% of the trials could have been feasibly pursued using currently available real-world data from structured EHRs and insurance claims. In particular, replication of the trials of FDA-regulated pharmaceutical interventions was often precluded by the lack of FDA approval at the time of trial publication. Regardless, our estimate was likely a best-case estimate; additional work would be needed to ultimately establish the reliability of observational data sources to generate RWE that could be sufficiently consistent with the trial’s intent. Because most trials published in the highest-impact journals were of new drugs that had not yet been approved by the FDA, the findings may reflect that these journals are more likely to publish the pivotal trials that support a new FDA approval.

However, the findings also reinforce the caution about the promise of RWE replacing RCTs. Using observational methods and data sources is not feasible for examining the safety and efficacy of a medical product that is not in widespread use in routine clinical practice.

This study also raises a second caution about the promise of RWE augmenting RCTs. Many of the trials could not be replicated because they would require data that are unlikely to appear in an EHR in structured form if at all. Several improvements in the collection and analysis of EHR data may enhance the use of real-world data to generate RWE, including increased use of patient-reported outcomes as part of routine clinical care, more widespread use of medical device surveillance initiatives,^14^ and improvements in natural language processing for free text in EHRs.^15^

The pharmaceutical industry spent $22.4 billion on phase 3 and phase 4 clinical trials in 2011,^16^ and the total cost of research and development for a single drug has been estimated at $2.9 billion.^17^ Thus, the theoretical potential for financial savings through RWE-based medical product evaluations is substantial. Furthermore, the evidentiary gaps and unanswered clinical questions that exist are unlikely to be pursued through clinical trials, but for which RWE may offer critical insights. For instance, RWE could be generated as postapproval evidence of medical product safety and efficacy for drugs with limited evidence to support their use.^18^ Similarly, producing sufficient evidence to support an FDA action to communicate a safety concern to patients and clinicians can take more than 4 years.^19^ Real-world evidence can be used to more rapidly generate actionable safety evidence, which is rarely pursued through clinical trials. Furthermore, RWE may be particularly useful because prospective postapproval studies can be difficult to recruit for given that patients can receive the therapy outside the trial, as occurred with the off-label use of atrial septal closure devices.^20,21^

The FDA continues to invest in its ability to use RWE. As part of the President’s Budget for fiscal year 2019, the FDA developed a $100 million proposal to build a medical data enterprise system using EHR data from about 10 million people to build a foundation for more robust postmarketing studies.^22^ This investment represents an evolution for the FDA from primarily relying on insurance claims for postmarketing research to using EHR data, which are a richer source of real-world clinical data and have a shorter reporting lag time than claims data. The proposal also aims to address the lack of standardization of structured EHR data and of interoperability between different systems. In addition, the RWE program of the FDA will develop a framework for evaluating studies that use RWE in terms of the reliability of real-world data sources and the ability of study designs and conducts to meet regulatory requirements.^23^ These initiatives could present uses for real-world data that may not have been considered otherwise.

### Limitations

This study has several limitations. First, the results are limited to a subset of all clinical trials published in 1 year, although we suspect that these trials represent those that have great implications for clinical practice and that are relevant to clinical practice guidelines and regulators. Second, in establishing whether an intervention could be ascertained from real-world data, we assumed that a pivotal trial for a new indication of a previously FDA-approved drug was replicable because it could theoretically be used off-label, although we did not confirm how common this practice was for each drug and indication. Third, in the analysis of study inclusion and exclusion criteria, we included trials if 80% of the individual criteria were considered as able to be ascertained from structured EHR or claims data, but we did not consider the relative importance or quality of each criterion. Fourth, we considered only primary end points mentioned in the article abstract and thus did not consider all measured study end points, limiting the generalizability of our findings. Fifth, we assumed that an EHR or claims diagnosis would be equivalent to a diagnosis in a clinical trial, which may not always be true given that clinical trials often follow stricter guidelines for diagnosis.^24,25^ For example, in a clinical trial, a diagnosis of myocardial infarction may require specific symptoms and troponin-level patterns, which were not evaluated for feasibility to be ascertained unless they were explicitly stated in the inclusion and exclusion criteria.

In addition, the definition of the feasibility of a trial’s characteristics to be ascertained from observational data does not consider whether an observational database would have a sufficient sample with which to perform an analysis (eg, a newly marketed drug or drug for a rare disease) or whether a financial incentive exists to use observational data to replicate a particular clinical trial. Thus, a trial that we identified to be replicable with real-world data may be only suitable to assess sample size feasibility within a given observational data source and then may be found not to be sufficiently powered or feasible to replicate. In addition, the determination of replicability inherently involved an element of subjectivity and did not consider natural language processing as a mechanism for extracting and analyzing real-world data. Although this method has been used in single-center studies, to our knowledge, it is not yet widely applied across health systems for the purpose of generating RWE. As methods become more advanced, opportunities to generate RWE and thereby use RWE to replicate trials will increase.

This study did not consider that the clinical settings in which real-world data are collected are often not the same as the environments in which RCTs are conducted. For instance, patient age, sex, race/ethnicity, or socioeconomic background; clinician location (eg, tertiary center vs private practice); or practice volume are likely to vary between the 2 settings. Nevertheless, this difference is one potential advantage of real-world data because the populations studied are more likely to be representative of the broader population of patients with disease.

## Conclusions

Although the potential to use real-world data for RWE is substantial, the current ability to replicate the design elements from clinical trials published in the highest-impact journals may be limited. Only 15% of the 220 US-based clinical trials published in high-impact journals in 2017 could have been feasibly replicated using currently available observational data from EHRs and insurance claims. These findings suggest the potential for observational data to complement but not completely replace RCTs. Further development of observational methods and data systems may help realize the potential of RWE and may, in turn, translate into more generalizable medical research and more rapid evaluation of medical products.

## Notes

Supplement. **eTable.** Proportion of Clinical Trials Published in High-Impact Journals in 2017 for Which the Specified Features Can Be Reliably Ascertained From Electronic Health Record or Claims Data, Stratified by Journal
