# Supplementary material for: Feasibility of Using Real-World Data to Replicate Clinical Trial Evidence
Source: JAMA Netw Open. 2019 Oct 9;2(10):e1912869. doi: 10.1001/jamanetworkopen.2019.12869 (PMC6802419; doi:10.1001/jamanetworkopen.2019.12869)
Supplement: Supplement. — eTable. Proportion of Clinical Trials Published in High-Impact Journals in 2017 for Which the Specified Features Can Be Reliably Ascertained From Electronic Health Record or Claims Data, Stratified by Journal [file jamanetwopen-2-e1912869-s001.pdf]

Supplementary Online Content

Bartlett VL, Dhruva SS, Shah ND, Ryan P, Ross JS. Feasibility of using real-world data to replicate clinical trial evidence. *JAMA Netw Open*. 2019;2(10):e1912869. doi:10.1001/jamanetworkopen.2019.12869

eTable. Proportion of Clinical Trials Published in High-Impact Journals in 2017 for Which the Specified Features Can Be Reliably Ascertained From Electronic Health Record or Claims Data, Stratified by Journal

This supplementary material has been provided by the authors to give readers additional information about their work.

© 2019 Bartlett VL et al. *JAMA Network Open*

eTable. Proportion of Clinical Trials Published in High-Impact Journals in 2017 for Which the Specified Features Can Be Reliably Ascertained From Electronic Health Record or Claims Data, Stratified by Journal

	**NEJM, No. (%) (n=112)**	**Lancet, No. (%) (n=28)**	**JAMA, No. (%) (n=44)**	**Others, No. (%) (n=36)**
Intervention can be ascertained from EHR or claims data;	50 (45)	12 (43)	15 (34)	9 (25)
And indication can be ascertained from EHR or claims data;	36 (32)	6 (21)	12 (27)	8 (22)
And 80% of study inclusion/exclusion criteria can be ascertained from EHR or claims data;	29 (26)	4 (14)	8 (18)	4 (11)
And at least one primary endpoint can be ascertained from EHR or claims data	23 (21)	3 (11)	4 (9)	3 (8)

© 2019 Bartlett VL et al. *JAMA Network Open*
